# Effect of Resveratrol on Hematological and Biochemical Alterations in Rats Exposed to Fluoride

**DOI:** 10.1155/2014/698628

**Published:** 2014-06-05

**Authors:** Nurgül Atmaca, Ebru Yıldırım, Bayram Güner, Ruhi Kabakçı, Fatih Sultan Bilmen

**Affiliations:** ^1^Department of Physiology, Faculty of Veterinary Medicine, Kirikkale University, 71451 Kirikkale, Turkey; ^2^Department of Pharmacology and Toxicology, Faculty of Veterinary Medicine, Kirikkale University, 71451 Kirikkale, Turkey; ^3^Department of Biochemistry, Faculty of Veterinary Medicine, Kirikkale University, 71451 Kirikkale, Turkey

## Abstract

We investigated the protective effects of resveratrol on hematological and biochemical changes induced by fluoride in rats. A total of 28 rats were divided into 4 groups: control, resveratrol, fluoride, and fluoride/resveratrol (*n* = 7 each), for a total of 21 days of treatment. Blood samples were taken and hematological and biochemical parameters were measured. Compared to the control group, the fluoride-treated group showed significant differences in several hematological parameters, including decreases in WBC, RBC, and PLT counts and neutrophil ratio. The group that received resveratrol alone showed a decrease in WBC count compared to the control group. Furthermore, in comparison to the control group, the fluoride group showed significantly increased ALT enzyme activity and decreased inorganic phosphorus level. The hematological and biochemical parameters in the fluoride + resveratrol treated group were similar to control group. In the fluoride + resveratrol group, resveratrol restored the changes observed following fluoride treatment, including decreased counts of WBC, RBC, and PLT, decreased neutrophil ratio and inorganic phosphorus levels, and elevated ALT enzyme activity. The present study showed that fluoride caused adverse effects in rats and that resveratrol reduced hematological and biochemical alterations produced by fluoride exposure.

## 1. Introduction

Fluoride is an essential element [1] but one that can cause serious health problems when drinking water contains fluoride at a concentration greater than 1 ppm or in the regions where a large amount of fluoride is released due to the burning of fluoride-loaded coal [2, 3]. Fluoride causes adverse effects in soft tissues such as blood, brain, and liver [4] by passing through the cell membrane [5]. Fluoride accumulates in bone tissues, where it can negatively affect hematopoiesis occurring in bone marrow [6]. In addition, as a site of very active metabolism, the liver is especially susceptible to fluoride toxicity [7]. However, it has been reported that changes due to fluoride exposure occur earlier in blood compared to other tissues and organs [8]. Previous studies revealed that fluoride had unfavorable effects on hematological and biochemical parameters [9, 10] in rats [11, 12], mice [13–15], sheep [16], rabbits [17, 18], dogs [19], camels [20], and humans [8, 21].

It has been reported that antioxidants in the diet, such as black tea extract [22], a combination of vitamin E with methionine and l-carnosine [12],* Panax* ginseng [14], and pineal proteins and melatonin [23], reduce the harmful effects of fluoride. Resveratrol (trans-3,5,4′-trihydroxystilbene) is a phytoalexin that is found in foods including grapes, plums, cranberries, and peanuts [24]. In addition, its antioxidant efficacy has been demonstrated in traumatic brain injury [25, 26], methotrexate-induced liver toxicity [27, 28], cisplatin- [29] and gentamicin-induced nephrotoxicity [30], and doxorubicin-induced cardiotoxicity [31]. However, no previous study exists on the potential of resveratrol to alleviate or eliminate adverse effects produced by fluoride exposure in rats.

The present study investigated the protective effects of resveratrol, a potent antioxidant, on fluoride-induced alterations in hematological and biochemical parameters of the blood, which is a target tissue for fluoride toxicity.

## 2. Materials and Methods

### 2.1. Drugs and Reagents

Sodium fluoride (NaF) was purchased from Merck.* trans*-Resveratrol was obtained from Cayman Chemical Company. All other chemicals were obtained from Merck Chemical, Inc. (Darmstadt, Germany).

### 2.2. Animals and Treatment

Experiments were carried out in male Wistar albino rats weighing 180–200 g, which were fed standard chow diet and water available ad libitum. The animals were housed in plastic cages, under a 12 h light/dark cycle (lights on from 08:00 a.m.) at a constant temperature of 25 ± 2°C with 42 ± 5% relative humidity. The study protocol was in accordance with the guidelines for animal research and it was approved by the Ethical Committee of the Kırıkkale University (10/155). Twenty-eight rats were randomly divided into four groups of seven animals each. Experimental groups were designed as follows: control group received distilled water; resveratrol group received daily resveratrol (12.5 mg/kg b.w.) intraperitoneally (i.p.) and distilled water fluoride group received daily 100 mg/L fluoride in drinking water; fluoride + resveratrol group received daily 100 mg/L fluoride in drinking water plus resveratrol (12.5 mg/kg b.w., i.p.) for 21 days. The dose and route of fluoride were chosen from previous studies [32, 33]. The selected dose and route for resveratrol used in the study were determined according to Mokni et al. [34].

### 2.3. Sample Collection

At the end of the 21st day, blood samples were collected into heparinised tubes by cardiac puncture from all animals, under light ether anesthesia.

### 2.4. Hematological Assay

White blood cells (WBC), lymphocyte and neutrophil ratio, red blood cells (RBC), hematocrit (Hct), hemoglobin (Hb), mean cell volume (MCV), mean cell hemoglobin (MCH), mean cell hemoglobin concentration (MCHC), and platelet count (PLT) were measured on Hematology Analyzer (Abacus Junior Vet 5, Austria).

### 2.5. Biochemical Assay

Cholesterol, albumin, calcium (Ca), and inorganic phosphate (Pi) levels were determined using Diasis (Germany) kits and aspartate aminotransferase (AST) (EC 2.6.1.1), alanine aminotransferase (ALT) (EC 2.6.1.2), and alkaline phosphatase (ALP) (EC 3.1.3.1) activities were measured using Biolabo (France) kits by spectrophotometer (Shimadzu, UV 1700, Shimadzu, Japan) using commercial assay kits according to the manufacturer's directions.

### 2.6. Statistical Analysis

Data processing was performed with the SPSS 15.0 (SPSS, Inc., Chicago, IL, USA). The normality of all data was assessed by Spreads versus Level with Levene Test. The hematological parameters were distributed nonparametrically and therefore tested using Kruskal-Wallis test followed by the Mann-Whitney *U* test to determine which of the four groups differed from each other. Biochemical parameters were analysed by one-way analysis of variance (ANOVA). When the *F* values were significant, Duncan's Multiple Range Test was performed. *P* values less than 0.05 were considered significant for all statistical calculations.

## 3. Results

As shown in Table 1, the data showed that treatment with fluoride caused a significant decrease in WBC, RBC, and PLT counts and neutrophil ratio in fluoride treated group as compared to control group. The hematological parameters in the fluoride + resveratrol treated group were similar to control group. In the fluoride plus resveratrol group, resveratrol alleviated the adverse effects on WBC, RBC, and PLT counts and neutrophil ratio caused by fluoride.

The biochemical parameters of the experimental groups were presented in Table 2. Enzyme activity of ALT was statistically increased in fluoride group as compared to control group. The plasma inorganic phosphorus level was significantly decreased in fluoride group as compared to control group. The biochemical parameters in the fluoride + resveratrol treated group were similar to control group. In the fluoride plus resveratrol group, resveratrol alleviated the adverse effects on ALT enzyme activity (*P* > 0.05) and inorganic phosphorus levels (*P* < 0.05) by fluoride. Other parameters did not differ in between.

## 4. Discussion

The present study investigated the protective effects of resveratrol on hematological and biochemical changes in rats induced by sodium fluoride. Similar to our studies, others have shown decreases in WBC and PLT counts [35], neutrophil ratio [20], and RBC count [13, 33, 36] in animals treated with fluoride. Some studies reported that fluoride caused changes in blood parameters such as Hb, Hct, MCV, MCH, and MCHC [12, 37], but in this study no statistical differences were observed in these parameters. In the present study, it was observed that fluoride-induced hematological changes were ameliorated in the group that received fluoride and resveratrol. It has been reported that fluoride toxicity causes hematopoietic progenitor cells injury in humans [6] and mice [38]. Decreased WBC, PLT, and neutrophil counts observed in the present study were probably due to harmful effects of fluoride on bone marrow and hematopoietic organs. In the present study, in which no anemia was observed, decreased RBC count may have been associated with a decreased rate of erythrogenesis due to the negative effect of fluoride on erythropoiesis or to shortened life span of erythrocytes and membrane degeneration by means of fluoride causing erythrocytes to change into echinocytes [39]. Unlike Juan et al. [40], who reported that high-dose resveratrol administration did not change hematological parameters in rats, the present study found WBC count to be significantly lower in the group that received resveratrol alone, as compared to the control group. Considering the anti-inflammatory characteristic of resveratrol [41], the observed decrease in WBC was expected. Hişmioğulları et al. [42] also reported that WBC count was decreased in rats given resveratrol. One of the most important findings of this study was that some fluoride-induced hematological changes were improved in the group that received resveratrol with fluoride. These results suggest a beneficial effect of resveratrol treatment against fluoride-induced changes in bone marrow and hematopoietic progenitor cells.

Some biochemical parameters, such as ALT activity, which is correlated with hepatic necrosis in rats [43] and indicates alteration in hepatic functions, were higher in the group that received fluoride as compared to the control group, despite the absence of differences in plasma ALP activity, urea, albumin, calcium, and inorganic phosphorus levels. The present study observed statistically insignificant increases in AST activity in the fluoride-treated group. Fluoride-induced elevations in AST and ALT activity have been reported in rats by Eraslan et al. [32] and in mice by Bouaziz et al. [44]. In contrast to the present study, Xiong et al. [45] reported that AST and ALT activities did not change as a result of exposure to fluoride in children diagnosed with dental fluorosis, whereas Kanbur et al. [10] reported that fluoride decreased ALT activity in mice, while increasing AST activity. Although there was no significant alteration in ALT or AST enzyme activity in the group that received resveratrol with fluoride, as compared to the control group and fluoride group, it was observed that both enzyme activities in this group showed a downward tendency. The liver, which has an active metabolism, is extremely sensitive to fluoride toxicity [46]. The insignificant decrease in AST and ALT enzyme activity in the group that received resveratrol with fluoride suggested that resveratrol had a protective effect on the liver.

An insignificant decrease in calcium level was recorded in the fluoride-treated group in this study. Phosphorus enters the cell as inorganic phosphorus via a secondary active transport mechanism [47]. The Na^+^/K^+^ ATPase pump, which is involved in the transport of phosphorus, has been reported to be inhibited by sodium fluoride [48]. Furthermore, Anderson et al. [49] demonstrated that fluoride increased Na^+^/K^+^ ATPase pump activity in osteoblast-like cells. Reduced inorganic phosphorus in the fluoride-treated group can be attributed to the increased activity of the Na^+^/K^+^ ATPase pump and the increase of inorganic phosphorus influx into the cell, which caused a decrease in phosphorus in the blood. In this study, the reduced inorganic phosphorus level was ameliorated by resveratrol.

## 5. Conclusions

In conclusion, two remarkable results have emerged from this study. First, fluoride exposure caused changes in hematological and biochemical parameters, which were not in perfect agreement with other studies. These discrepancies are thought to be related to the dose and duration of fluoride exposure, as well as to differences in animal species and individual differences. Second, resveratrol reduced some harmful effects induced by fluoride treatment.

## Figures and Tables

**Table 1:** Hematological parameters in control and experimental groups.

	Control	Resveratrol	Fluoride	Fluoride + resveratrol	*P* values
WBC (10^3^/mm^3^)	7.99^a^	4.22^bc^	4.85^c^	8.15^a^	0.002
Lymphocyte (%)	64.20	61.80	71.90	53.50	NS
Neutrophil (%)	32.60^a^	32.30^a^	22.80^b^	39.10^a^	0.029
RBC (10^6^/mm^3^)	7.99^a^	8.71^a^	7.46^b^	8.05^a^	0.022
HGB (gr/dL)	13.70	13.10	13.00	12.00	NS
HCT (%)	38.95	35.96	38.87	34.59	NS
MCV (fL)	49.00	45.00	52.00	43.00	NS
MCH (pg)	17.30	15.40	17.10	14.90	NS
MCHC (g/dL)	34.00	34.70	32.90	34.00	NS
PLT (10^3^/mm^3^)	758^ac^	999^a^	403^b^	699^c^	0.007

Results were expressed as median. Data having different superscript letter within the same row were statistically different from each other. NS: not significant.

**Table 2:** Plasma biochemical constituents in control and experimental groups.

	Control	Resveratrol	Fluoride	Fluoride + resveratrol	*P* values
ALT (U/L)	10.25 ± 3.72^b^	11.86 ± 2.25^b^	33.58 ± 6.73^a^	23.72 ± 4.76^ab^	0.006
AST (U/L)	95.01 ± 11.91	84.62 ± 9.86	112.34 ± 9.95	94.84 ± 4.72	NS
ALP (U/L)	399.10 ± 88.83	290.64 ± 18.05	385.12 ± 41.22	338.46 ± 29.43	NS
Cholesterol (mg/dL)	41.81 ± 3.29	46.79 ± 5.34	46.27 ± 2.00	56.72 ± 3.74	NS
Albumin (g/dL)	2.92 ± 0.16	2.56 ± 0.20	2.64 ± 0.20	2.66 ± 0.21	NS
Ca (mg/dL)	10.24 ± 0.67	9.85 ± 0.71	8.12 ± 0.44	8.50 ± 0.64	NS
Pi (mg/dL)	8.62 ± 0.32^a^	8.79 ± 0.74^a^	4.64 ± 0.65^c^	6.45 ± 0.31^b^	0.000

The data were expressed as mean ± standard error. Data having different superscript letter within the same row were statistically different from each other. NS: not significant.
